# Long term prognostic implication of newly detected abnormal glucose tolerance among patients with stable cardiovascular disease: a population-based cohort study

**DOI:** 10.1186/s12967-021-02950-y

**Published:** 2021-06-30

**Authors:** Maryam Kabootari, Samaneh Asgari, Seyedeh Maryam Ghavam, Hengameh Abdi, Fereidoun Azizi, Farzad Hadaegh

**Affiliations:** 1 Metabolic Disorders Research Center, Golestan University of Medical Sciences, Gorgan, Iran; 2 Prevention of Metabolic Disorders Research Center, Research Institute for Endocrine Sciences, Shahid Beheshti University of Medical Sciences, No. 24, Yamen Street, Velenjak, P.O. Box: 19395-4763, Tehran, Iran; 3 Department of Internal Medicine, Taleghani Educational Hospital, School of Medicine, Shahid Beheshti University of Medical Sciences, Tehran, Iran; 4 Endocrine Research Center, Research Institute for Endocrine Sciences, Shahid Beheshti University of Medical Sciences, Tehran, Iran

**Keywords:** Impaired fasting glucose, Impaired glucose tolerance, Newly diagnosed diabetes, Cardiovascular disease

## Abstract

**Background:**

Fasting plasma glucose (FPG) and 2-h post challenge plasma glucose (2 h-PCPG), whether as continuous or categorical variables, are associated with incident cardiovascular disease (CVD) and diabetes; however, their role among patients with existing CVD is a matter of debate. We aimed to evaluate associations of different glucose intolerance states with recurrent CVD and incident diabetes among subjects with previous CVD.

**Methods:**

From a prospective population-based cohort, 408 Iranians aged  ≥  30 years, with history of CVD and without known diabetes were included. Associations of impaired fasting glucose (IFG) according to the American Diabetes Association (ADA) and World Health Organization (WHO) criteria, impaired glucose tolerance (IGT), newly diagnosed diabetes (NDM) with outcomes of interest were determined by multivariable Cox proportional hazard models after adjustment for traditional risk factors. Furthermore, FPG and 2 h-PCPG were entered as continuous variables.

**Results:**

Over a decade of follow-up, 220 CVD events including 89 hard events (death, myocardial infarction and stroke) occurred. Regarding prediabetes, only IFG-ADA was associated with increased risk of hard CVD [hazard ratio(HR), 95%CI: 1.62,1.03–2.57] in the age-sex adjusted model. In patients with NDM, those with FPG ≥ 7 mmol/L were at higher risk of incident CVD/coronary heart disease(CHD) and their related hard outcomes (HR ranged from 1.89 to 2.84, all P < 0.05). Moreover, those with 2 h-PCPG ≥ 11.1 mmol/L had significant higher risk of CVD (1.46,1.02–2.11), CHD (1.46,1.00–2.15) and hard CHD (1.95:0.99–3.85, P = 0.05). In the fully adjusted model, each 1 SD increase in FPG was associated with 20, 27, 15 and 25% higher risk of CVD, hard CVD, CHD and hard CHD, respectively; moreover each 1 SD higher 2 h-PCPG was associated with 21% and 16% higher risk of CVD, and CHD, respectively. Among individuals free of diabetes at baseline (n = 361), IFG-ADA, IFG-WHO and IGT were significantly associated with incident diabetes (all P < 0.05); significant associations were also found for FPG and 2 h-PCPG as continuous variables (all HRs for 1-SD increase > 2, P < 0.05).

**Conclusions:**

Among subjects with stable CVD, NDM whether as high FPG or 2 h-PCPG, but not pre-diabetes status was significantly associated with CVD/CHD and related hard outcomes.

**Supplementary Information:**

The online version contains supplementary material available at 10.1186/s12967-021-02950-y.

## Introduction

Cardiovascular disease (CVD) is one of the high-burden diseases in the Middle East and North Africa (MENA) region and specially among Iranian population [1]. Individuals with history of CVD are at high risk of recurrent CVD events; traditional CVD risk factors, the number of stenotic coronaries, the presence of heart failure (HF), atrial fibrillation, cardiovascular treatment and geographic region have been reported as main determinants in international models for predicting recurrent CVD [2, 3].

Our previous study among patients with established CVD showed that type 2 diabetes is associated with > twofold higher risk of recurrent CVD events [4]. However, the impact of intensive glucose control versus appropriate management of blood pressure and lipid according to the guidelines on prevention of recurrent CVD in patients with diabetes are still inconclusive [5]. Impaired fasting glucose (IFG), impaired glucose tolerance (IGT) and newly diagnosed diabetes mellitus (NDM) with a high incidence rate among Iranian population [6, 7], are common disorders in patients with CVD [8]. Associations between prediabetes and NDM with recurrent CVD have been assessed in some short- and long-term hospital-based studies with inconsistent findings [9–12]; These studies were performed among patients with history of myocardial infarction (MI), coronary artery bypass graft (CABG), percutaneous coronary intervention (PCI), HF [13] or history of acute coronary syndrome (ACS). According to these studies, Ryden et al. [14] strongly recommend using oral glucose tolerance test (OGTT) for all patients with coronary artery disease (CAD) without known dysglycemia to improve the prediction of recurrent CV events. However, recently, the investigators of ARTEMIS study [9] examined the prognostic significance of prediabetes among CAD patients in the stable phase of CAD. Findings showed that the presence of prediabetes, regardless of its definition, was not associated with higher incidence of major adverse cardiovascular events (MACE). To the best of our knowledge, this controversial issue has poorly been addressed in population-based studies, especially in regions with high burden of CVD [1].

In the current study, we aimed to investigate the associations between FPG and 2 h-post challenge plasma glucose (2 h-PCPG), whether as continuous or categorical variables, with subsequent CVD/coronary heart disease (CHD) events and related hard outcomes as well as incident type 2 diabetes among Iranian subjects with stable CVD and without known diabetes from the Tehran Lipid and Glucose Study (TLGS), the oldest population-based cohort of MENA region.

## Materials and methods

### Study population

The present study was conducted within the framework of the TLGS, an ongoing large prospective community-based study of a representative urban sample of Tehranian population with the aim of determining the prevalence and incidence of non-communicable diseases and related risk factors. Tehran is an ethnically diverse city. Population of Tehran comprises numerous ethnic, religious and linguistic groups, prominently including Persians, Azeris, Kurds, Lurs, Arabs, Baluchis, and Turkmen; 75% of people in Tehran identify themselves as Persian [15].

Briefly, participants have been recruited in first (1999–2001) and second (2002–2005) phases and follow-up visits has continued at approximately 3-year intervals, i.e. the third phase: 2005–2008, the fourth phase: 2009–2011, the fifth phase: 2012–2015 and the sixth phase: 2015–2018. Details of study design, sampling frame and rationale have been explained previously [16]. All participants gave written informed consents according to the Helsinki Declaration guideline and the study was approved by the local ethics committee (medical ethics committee of the Research Institute for Endocrine Sciences).

### Outcome 1: recurrent CVD/CHD

**Fig. 1:**
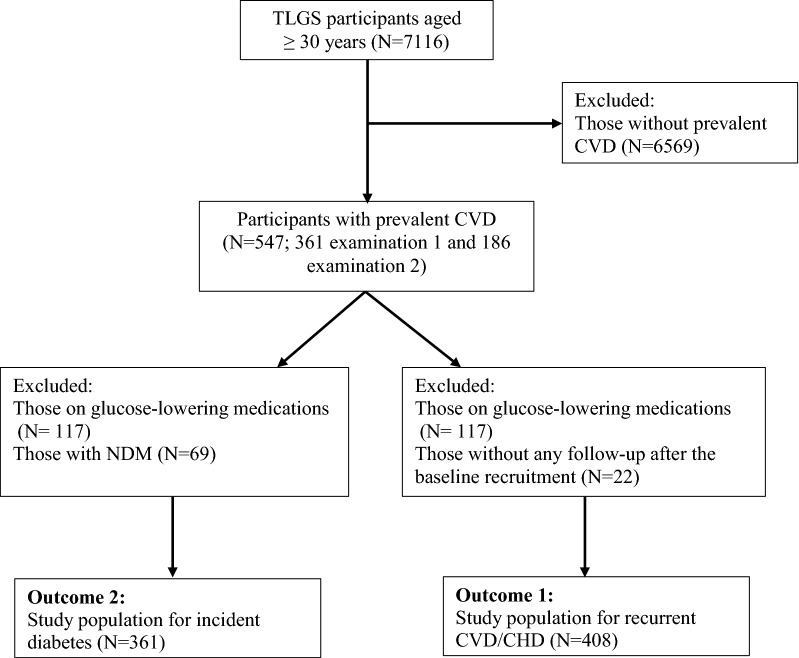
Flowchart of the study population. *TLGS* Tehran Lipid and Glucose Study, *CVD* cardiovascular disease, *CHD* coronary heart disease, *NDM* newly diagnosed diabetes mellitus

In the present study, among 7116 participants aged ≥ 30 years, 547 participants with prevalent CVD were included [361 individuals from the study baseline (1999–2002) and 186 ones from the second phase (2002–2005)]. After excluding those with known diabetes (i.e. those taking glucose-lowering medications at baseline visit, n = 117), and those without any follow-up after the baseline recruitment (n = 22), 408 participants remained for the current study and were followed until March 2016 (overall response rate: 408/430 = 95%) (Fig. 1).

### Outcome 2: type 2 diabetes

In the same data set, when considering incident diabetes as outcome, from the total of 547 participants with prevalent CVD, those on glucose-lowering medications at the baseline visit (n = 117) and those with NDM (n = 69) were excluded and 361 participants entered for data analysis (Fig. 1).

### Clinical and laboratory measurements

Demographic information, medical history, smoking habits and history of CVD were obtained from participants during interviews, using a validated questionnaire at baseline and each follow-up. Details of anthropometric measurements including weight, height and waist circumferences (WC) have been described elsewhere [16]. Body mass index (BMI) was calculated as weight in kilograms divided by square of height (m^2^). Blood pressure (BP) was measured using a standardized mercury sphygmomanometer (calibrated by the Iranian Institute of Standards and Industrial Researches), twice on the right arm in a seated position after at least 15-min rest and the mean of these two measurements was considered as the participant’s BP.

Blood samples were taken between 7:00 and 9:00 AM after 12–14 h overnight fasting and a standard oral glucose tolerance test using 75 g glucose for those without history of taking glucose-lowering medications was performed. Details about measurements of serum glucose, total cholesterol (TC), triglycerides (TG), and high-density lipoprotein cholesterol (HDL-C) have been previously reported [16].

### Definition of terms

For categorization of glucose tolerance status, we used both American Diabetes Association (ADA) [17] and World Health Organization (WHO) [18] criteria as follows: normal fasting glucose (NFG)-5.6: FPG < 5.6 mmol/L, NFG-6.1: FPG < 6.1 mmol/L, normal glucose tolerance (NGT): 2 h-PCPG < 7.8 mmol/L, IFG-ADA: 5.6 ≤ FPG < 7 mmol/L, IFG-WHO: 6.1 ≤ FPG < 7 mmol/L and, IGT: 7.8 ≤ 2 h-PCPG < 11 mmol/L. NDM was defined as FPG ≥ 7.0 mmol/L or 2 h-PCPG ≥ 11.0 mmol/L at the first visit among those without history of taking glucose-lowering medications.

History of CVD was defined as history of ACS, definite coronary artery disease according to angiography results (> 50% stenosis in at least one major coronary vessel), non-fatal MI, non-fatal stroke, CABG and PCI.

Positive family history of premature CVD was defined as history of CHD or stroke in a male first-degree relative aged < 55 years or a female first-degree relative aged < 65 years. Positive family history of diabetes was determined as having at least a first-degree relative with diabetes. Smoking status was described as current smoker versus non-smoker. Hypertension was defined as systolic BP ≥ 140 mmHg and/or diastolic BP ≥ 90 mmHg or using anti-hypertensive drugs. Hypercholesterolemia was described as TC levels ≥ 5.1 mmol/L and/or using lipid lowering medications. Hypertriglyceridemia was defined as TG ≥ 1.69 mmol/L and low HDL-C as HDL-C < 1.06 mmol/L and < 1.29 mmol/L in men and women, respectively [19].

### Outcomes

Detailed description of outcome data collection has been published previously [20]. Each participant in the TLGS is followed-up by telephone call from a trained nurse for any medical event leading to hospitalization during the past year; thereafter, a trained physician collects complementary data regarding that event during a home or hospital visit. In the case of mortality, data are collected from the hospital or death certificate by an authenticated local physician. Collected data is then evaluated by an outcome committee blinded to the status of baseline risk factors including the principal investigator, an internist, an endocrinologist, a cardiologist, an epidemiologist and other experts if needed to assign a specific outcome for every event.

In the current study, CHD events included cases of 1) definite MI [positive electrocardiogram (ECG) and biomarkers including creatine kinase (CK), CK-MB, troponin and myoglobin], 2) probable MI (positive ECG findings plus cardiac symptoms or signs and normal or equivocal biomarkers), 3) unstable angina pectoris (new cardiac symptoms or changing symptom patterns and positive ECG findings with normal biomarkers) 4) angiography-proven CHD, and 5) CHD death (any death in hospital due to CHD or sudden cardiac death caused by cardiac disease occurring  ≤ 1 h after beginning of symptoms) [21]. Furthermore, CVD was clarified as a composite of CHD and cerebrovascular events [transient ischemic attack (TIA), ischemic or hemorrhagic stroke and cerebrovascular death]. To assess the relationship of newly detected abnormal glucose tolerance with the more severe form of cardiovascular events, hard CHD events were defined as the occurrence of nonfatal MI and CHD death and hard CVD event considered as nonfatal MI, nonfatal stroke and CVD death [22].

### Statistical analysis

Little's Missing Completely at Random (MCAR) test was used to check whether or not the missing data follow a completely random pattern [23]. The result showed that the missingness is not completely at random (*P-value* < 0.001). Therefore, for dealing with missing values, we used multiple imputations by chained equations (MICE) with 10 imputed data sets since ≈10% of cases were incomplete (2 h-PCPG, BMI, and low physical activity: ≈10%; other covariates: < 3%) [24, 25]. We obtained all estimates by averaging results across the imputed datasets.

Baseline characteristics are expressed as mean [standard deviation (SD)] and median [interquartile range (IQR)] for continuous variables with and without normal distributions, respectively and number (%) for categorical ones across the NFG, IFG and NDM. To compare the baseline characteristics in different glucose intolerance categories, ANOVA (or Kruskal–Wallis for variables with non-normal distribution) and chi-square tests were employed for continuous and categorical variables, respectively.

**Fig. 2:**
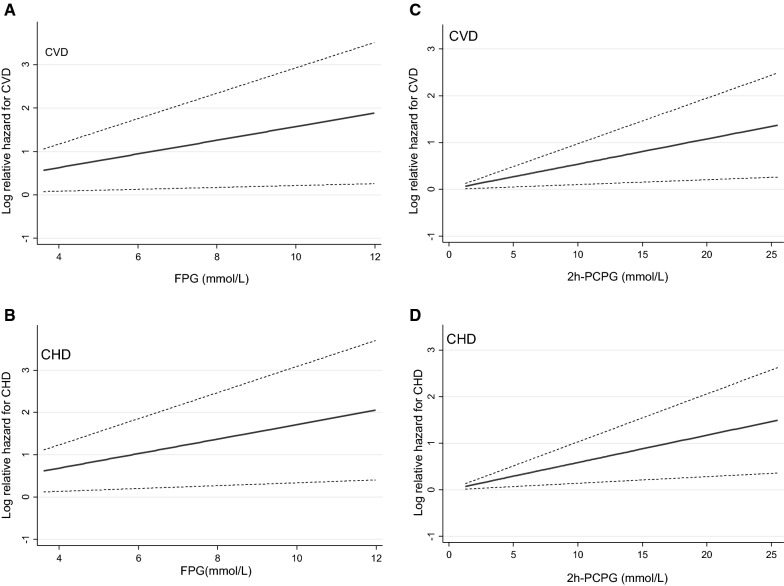
Regression cubic spline model for the associations of FPG with (**A**) cardiovascular disease (CVD) and (**B**) coronary heart disease (CHD) as well as 2 h-PCPG with (**C**) CVD and (**D**) CHD

To be able to capture a potential nonlinear association between FPG/2 h-PCPG and incident CVD/CHD outcomes, restricted cubic splines with 4 knots defining the 5th, 25th, 75th, and 95th percentiles, were used. As shown in Fig. 2, we accepted the null hypothesis that outcome risks were a linear function of the FPG/2 h-PCPG.

Multivariable Cox proportional hazard models were used to evaluate associations of the different glucose intolerance categories with recurrent CVD, hard CVD, CHD, hard CHD and incident diabetes separately, using NFG-5.6 or NFG-6.1 or NGT as reference.

The survival time for CVD, CHD, and the related hard outcomes was defined as the time between the entered date and the event date. Additionally, for the censored participants (leaving the residential area, death, loss to follow-up or end of follow-up until 20 March 2016), the survival time was defined as the difference between the entered date and the last available follow-up date.

Regarding incident diabetes, the event date was defined as the date of incident diabetes or being censored (leaving the residential area, death, loss to follow-up, or end of follow-up until 20 March 2018); the date of the event was defined as the mid-time between the last observation date of without and with diabetes. Additionally, for the censored participants, the censored date was defined as the difference between the last observation data without diabetes.

For the Cox regression analysis, two models were designed: model 1 included sex and baseline measurements of age; in model 2, following potential risk factors based on the literature review [26] were added for incident CVD/CHD: BMI, heart rate, family history of premature CVD (reference: no), hypertension (reference: no), high TC (reference: no), low HDL-C (reference: no), current smoking (reference: no), use of aspirin (reference: no), use of β-blocker (reference: no), and low physical activity (reference: no). Considering the outcome of incident diabetes, the literature review [27, 28] determined following potential risk factors to be included in model 2: BMI, family history of diabetes, low HDL-C (reference: no), high TG (reference: no), and low physical activity (reference: no).

The proportionality in the Cox model was evaluated with the Schoenfeld residual test and generally, all proportionality assumptions were appropriate. Statistical analysis was performed using STATA version 14 (Stata Corp LP, College Station, Texas) statistical software. P-values < 0.05 were considered statistically significant.

## Results

**Fig. 3:**
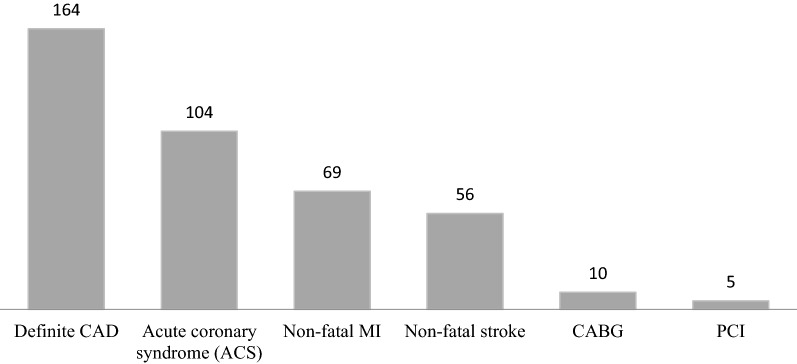
Details of previous events and interventions in subjects with cardiovascular disease at baseline visit. *CAD* coronary artery disease, *MI* myocardial infarction, *CABG* coronary artery bypass graft,* PCI* percutaneous coronary intervention

Previous CVD in 408 subjects consisted of history of definite CAD (164), ACS (104), non-fatal MI (69), non-fatal stroke (56) CABG (10) and PCI (5) (Fig. 3). Baseline characteristics of study participants in different glucose tolerance categories according to ADA criteria are shown in Table 1. The mean (SD) age of total population was 60.6 (10.5) years and 58.3% were men. Generally, there were significant differences between different groups of glucose tolerance in BMI, WC, SBP, FPG, 2 h-PCPG, TC, triglycerides and low physical activity levels.

**Table 1:** Baseline characteristics of the study population

	Total population (n = 408)	NFG (n = 253)	IFG- ADA (n = 118)	NDM(n = 37)	P-Value	Statistics ^b^
Continuous variables, mean (SD)						
Age (years)	60.6 (10.5)	60.0 (10.9)	61.3 (10.2)	62.8 (8.7)	0.22	1.51
BMI (kg/m^2^)	28.2 (4.7)	27.6 (4.6)	29.0 (4.8)	29.2 (4.8)	0.009	4.82
WC (cm)	97.1 (10.5)	95.6 (10.6)	99.2 (9.8)	100.7 (9.6)	0.001	7.54
SBP (mmHg)	130.7 (21.2)	128. 4 (20.3)	132.8 (20.7)	139.2 (25.6)	0.006	5.22
DBP (mmHg)	78.4 (11.8)	78.2(12.2)	78.1 (11.4)	80.2 (9.6)	0.62	0.48
HR (beats/minute)	76.7 (11.9)	75.7(12.0)	78.3 (12.1)	78.7 (9.9)	0.08	2.49
FPG (mmol/L)	5.6 (1.4)	5.0(0.4)	6.1 (0.4)	9.1 (2.1)	< 0.001	555.01
2 h-PCPG (mmol/L)	8.2 (4.3)	6.4(1.8)	8.9 (2.6)	18.2 (5.8)	< 0.001	323.8
TC (mmol/L)	5.4 (1.1)	5.3 (1.1)	5.4(1.0)	5.8 (1.4)	0.04	3.25
HDL-C (mmol/L)	0.98(0.2)	0.98(0.2)	0.97(0.3)	0.94 (0.3)	0.69	0.37
Triglycerides (mmol/L) ^a^	1.85(1.24)	1.8(1.2)	1.8(1.3)	2.4 (1.3)	0.04	4.23
Categorical variables, n (%)						
Gender (men)	238(58.3)	150(59.3)	64(54.2)	24(64.9)	0.47	1.56
Family history of CVD	62(15.2)	39(15.4)	19(16.1)	4(10.8)	0.79	0.51
Current smoking	69(16.9)	42(16.6)	20(16.9)	7(18.9)	0.94	0.12
Low physical activity	174(42.6)	114(45.1)	53(44.9)	7(18.9)	0.007	9.37
Hypertension	218(53.4)	131(51.8)	66(55.9)	21(56.8)	0.69	0.74
Hypercholesterolemia	245(60.0)	149(58.9)	73(61.9)	23(62.2)	0.86	0.37
Medications						
Anti-hypertensive medication	133(32.6)	83(32.8)	37(31.4)	13(35.1)	0.88	0.20
Lipid-lowering medication	45(11.0)	27(10.7)	13(11.0)	5(13.5)	0.83	0.27
Aspirin use	198(48.5)	129(51.0)	49(41.5)	20(54.1)	0.17	3.38

Values are expressed as mean (SD) or median (interquartile range) for continuous variables and n (%) for categorical variables

Missing values were imputed with multiple imputation. *NFG* normal fasting glucose, *IFG* impaired fasting glucose, *NDM* newly diagnosed diabetes, *ADA* American Diabetes Association, *BMI* body mass index, *WC* waist circumference, *SBP* systolic blood pressure, *DBP* diastolic blood pressure, *HR* heart rate, *FPG* fasting plasma glucose, *2 h-PCPG* 2 h post challenge plasma glucose, *TC* total cholesterol, *HDL-C* High density lipoprotein-cholesterol, *CVD* cardiovascular disease

^a^Median and interquartile range (IQR)

^b^ANOVA F statistics for continues variables and Pearson Chi-square or Fisher exact value for categorical variables. For Triglycerides Kruskal Wallis test was reported

Over a decade of follow-up, 220 CVD including 89 hard events and 202 CHD including 58 hard events occurred. Among 361 subjects with prevalent CVD and free of diabetes at baseline, 141 incident diabetes occurred with an incidence rate of 38.88 (32.97–45.86) per 1000 persons-years.

### Outcome 1: recurrent CVD/CHD

Risks of adverse cardiovascular outcomes based on different glucose intolerance categories are illustrated in Tables 2 and 3. Considering the level of FPG, subjects with IFG using both WHO and ADA definitions had no statistically significant higher risk of CVD/CHD even in the age and sex adjusted model. Regarding hard outcomes, only IFG-ADA was associated with significantly higher risk of hard CVD in model 1 [hazard ratio (HR), 95% CI 1.62, 1.03–2.57] and showed a signal for the event in model 2 (HR, 95% CI 1.52, 0.95–2.45, *p* = 0.08). Subjects with NDM using FPG definition, in both IFG-WHO and IFG-ADA datasets, were at > twofold higher risk of CVD (2.15, 1.41–3.27), hard CVD (2.41, 1.25–4.67), CHD (2.03, 1.30–3.16) and hard CHD (2.84, 1.31–6.19) compared to subjects with NFG in the fully adjusted model.

**Table 2:** Multivariable adjusted risks of CVD and hard CVD based on different glucose intolerance categories. (n = 408)

	CVD HR (95%CI)			Hard CVD HR (95%CI)		
	E/N	Model 1	Model 2	E/N	Model 1	Model 2
FPG-WHO (mmol/L)						
< 6.1	169/327	1.00	1.00	66/327	1.00	1.00
6.1–6.9	21/44	0.92 (0.58–1.45)	0.87 (0.55–1.39)	10/44	1.13 (0.58–2.21)	0.97 (0.49–1.93)
≥ 7	30/37	2.02 (1.36–3.00)	2.07 (1.39–3.12)	13/37	1.95 (1.07–3.53)	2.02 (1.07–3.80)
FPG-ADA (mmol/L)						
< 5.6	129/253	1.00	1.00	45/253	1.00	1.00
5.6–6.9	61/118	1.07 (0.79–1.46)	1.05 (0.77–1.44)	31/118	1.62 (1.03–2.57)	1.52 (0.95–2.45)
≥ 7	30/37	2.09 (1.40–3.12)	2.15 (1.41–3.27)	13/37	2.28 (1.23–4.22)	2.41 (1.25–4.67)
2 h-PCPG (mmol/L)						
< 7.8	123/240	1.00	1.00	50/240	1.00	1.00
7.8–11.0	54/105	1.01 (0.73–1.39)	0.97 (0.70–1.36)	20/105	0.93 (0.55–1.56)	0.90 (0.53–1.55)
≥ 11	43/63	1.48 (1.04–2.10)	1.46 (1.02–2.11)	19/63	1.59 (0.93–2.70)	1.54 (0.88–2.70)

Model 1: age and sex

Model 2: model 1+ body mass index, current smoking, family history of premature CVD, hypertension, hypercholesterolemia, low HDL-C, heart rate, use of aspirin, use of beta blockers and low physical activity.

*E* event in the target group, * N* total sample size in the target group, *CVD* cardiovascular disease, * CHD* coronary heart disease,* HR* hazard ratio, * CI* confidence interval, *FPG* fasting plasma glucose,* 2h-PCPG* 2h post challenge plasma glucose, *WHO* World Health Organization,* ADA* American Diabetes Association

**Table 3:** Multivariable adjusted risks of CHD and hard CHD based on different glucose intolerance categories. (n = 408)

	CHD HR (95%CI)			Hard CHD HR (95%CI)		
	E/N	Model 1	Model 2	E/N	Model 1	Model 2
FPG-WHO (mmol/L)						
< 6.1	156/327	1.00	1.00	41/327	1.00	1.00
6.1–6.9	19/44	0.89 (0.55–1.43)	0.83 (0.51–1.35)	7/44	1.27 (0.57–2.84)	1.01 (0.44–2.32)
≥ 7	27/37	1.89 (1.25–2.87)	1.97 (1.28–3.03)	10/37	2.31 (1.15–4.62)	2.56 (1.21–5.39)
FPG-ADA (mmol/L)						
< 5.6	120/253	1.00	1.00	30/253	1.00	1.00
5.6–6.9	55/118	1.04 (0.75–1.43)	1.02 (0.73–1.41)	18/118	1.39 (0.77–2.49)	1.30 (0.71–2.40)
≥ 7	27/37	1.94 (1.27–2.97)	2.03 (1.30–3.16)	10/37	2.50 (1.22–5.12)	2.84 (1.31–6.19)
2 h-PCPG (mmol/L)						
< 7.8	112/240	1.00	1.00	31/240	1.00	1.00
7.8–11.0	51/105	1.02 (0.73–1.42)	1.00 (0.70–1.40)	13/105	0.97 (0.51–1.85)	0.93 (0.47–1.83)
≥ 11	39/63	1.46 (1.01–2.11)	1.46 (1.00–2.15)	14/63	1.91 (1.01–3.60)	1.95 (0.99–3.85)

Model 1: age and sex

Model 2: model 1+ body mass index, current smoking, family history of premature CVD, hypertension, hypercholesterolemia, low HDL-C, heart rate, use of aspirin, use of beta blockers and low physical activity.

*E* event in the target group, * N* total sample size in the target group, *CVD* cardiovascular disease, * CHD* coronary heart disease,* HR* hazard ratio, * CI* confidence interval, *FPG* fasting plasma glucose,* 2h-PCPG* 2h post challenge plasma glucose, *WHO* World Health Organization,* ADA* American Diabetes Association

Regarding 2 h-PCPG, subjects with IGT had no significantly higher risk of CVD/CHD and related hard outcomes compared to those with 2 h-PCPG < 7.8 mmol/L. Moreover, NDM (2 h-PCPG ≥ 11.0 mmol/L) was significantly associated with risk of CVD (1.46, 1.02–2.11), CHD (1.46, 1.00–2.15) and hard CHD (1.95, 0.99–3.85, P = 0.05) in model 2.

As shown in Table 4, when FPG is considered as a continuous variable, HRs (95% CI) associated with a 1 SD increase in FPG were 1.20 (1.08–1.34) for CVD, 1.27 (1.07–1.51) for hard CVD, 1.15 (1.02–1.29) for CHD and 1.25 (1.00–1.56) for hard CHD in the fully adjusted model; the corresponding values for 2 h-PCPG were 1.21(1.07–1.36), 1.21 [(0.99–1.47), *p* = 0.06], 1.16 (1.03–1.32) and 1.23 (0.96–1.57), respectively. Moreover, when both FPG and 2 h-PCPG were entered in the same model, risk of CVD/CHD was not significant for these variables excluding for hard CVD events when higher values of FPG but not 2 h-PCPG was associated with marginally significant risk (Data not shown).

**Table 4:** Adjusted HR (95% CI) for adverse cardiovascular outcomes per 1-SD increase of FPG and 2 h-PCPG. (n = 408)

	CVD		Hard CVD		CHD		Hard CHD	
	HR (95%CI)	P-value	HR (95%CI)	P-value	HR (95%CI)	P-value	HR (95%CI)	P-value
FPG								
Model 1	1.21 (1.09–1.34)	< 0.001	1.27 (1.08–1.49)	0.004	1.15 (1.03–1.29)	0.01	1.22 (0.99–1.49)	0.06
Model 2	1.20 (1.08–1.34)	0.001	1.27 (1.07–1.51)	0.006	1.15 (1.02–1.29)	0.02	1.25 (1.00–1.56)	0.05
2 h-PCPG								
Model 1	1.21 (1.08–1.36)	0.001	1.21 (1.01–1.45)	0.04	1.17 (1.03–1.32)	0.01	1.20 (0.96–1.51)	0.11
Model 2	1.21 (1.07–1.36)	0.002	1.21 (0.99–1.47)	0.06	1.16 (1.03–1.32)	0.02	1.23 (0.96–1.57)	0.10

*CVD* cardiovascular disease, *CHD* coronary heart disease, *HR* hazard ratio, *CI* confidence interval, *FPG *fasting plasma glucose, *2 h-PCPG* 2 h post challenge plasma glucose

Model 1: age and sex

Model 2: model 1 + body mass index, current smoking, family history of premature CVD, hypertension, hypercholesterolemia, low HDL-C, heart rate, use of aspirin, use of beta blockers and low physical activity

### Outcome 2: incident diabetes

As shown in Table 5, both IFG-WHO [2.28 (1.44–3.63), *p* < 0.001] and IFG-ADA [2.37 (1.66–3.37), *p* < 0.001] were associated with significantly higher risk of incident diabetes in the fully adjusted model. Moreover, IGT was associated with > 2.5-fold higher risk in the fully adjusted model [2.67(1.87–3.79), *p* < 0.001]. HRs (95% CI) per each 1-SD increase in FPG and 2 h-PCPG for incident diabetes in the fully adjusted model were 9.90 [(4.67–20.98), *p* < 0.001] and 2.79 [(2.05–3.79), *p* < 0.001], respectively.

**Table 5:** Risks of incident diabetes based on different glucose intolerance states. (n = 361)

	HR	Model 1	P value	HR	Model 2	P value
		95%(CI)			95%(CI)	
FPG and 2 h-PCPG categories						
IFG-WHO	2.79	1.78–4.37	< 0.001	2.28	1.44–3.63	< 0.001
IFG-ADA	2.46	1.75–3.46	< 0.001	2.37	1.66–3.37	< 0.001
IGT	2.94	2.09–4.12	< 0.001	2.67	1.87–3.79	< 0.001
Per 1-SD increase						
FPG	12.82	6.18–26.60	< 0.001	9.90	4.67–20.98	< 0.001
2 h-PCPG	3.20	2.39–4.29	< 0.001	2.79	2.05–3.79	< 0.001

*HR* hazard ratio, *CI* confidence interval, *IFG* impaired fasting glucose, *IGT* impaired glucose tolerance, *WHO* World Health Organization, *ADA* American Diabetes Association, *FPG* fasting plasma glucose, *2 h-PCPG* 2 h post challenge plasma glucose

Model 1: age and sex

Model 2: model 1 plus family history of diabetes, body mass index, low physical activity, hypertriglyceridemia and low HDL-C

### Sensitivity analyses

To show the robustness of our findings, we additionally performed three sensitivity analyses. First, we repeated our analysis among subject with complete data (n = 301); the results were generally in line with the imputed dataset; however, in complete case analysis, NDM (FPG ≥ 7 mmol/L) was only associated with CVD/CHD but not hard outcomes and NDM (2 h-PCPG ≥ 11.0 mmol/L) was not a risk for any cardiovascular outcome. Moreover, each 1 SD increase in FPG and 2 h-PCPG was only associated with CVD/CHD (Additional file 1: Tables S1 and S2). Second, we analyzed data after exclusion of those with stroke at baseline (n = 56) and also in patients with definite CAD; results were generally in agreement with main findings (Additional file 1: Tables 3, 4 and 5). Third, to compare our findings with those of other studies, we defined abnormal glucose tolerance (AGT) as 2 h-PCPG ≥ 7.8 mmol/L versus 2 h-PCPG < 7.8 mmol/L (reference group). Accordingly, AGT similar to IGT was not associated with any cardiovascular outcome (Data not shown).

## Discussion

Findings of this population-based cohort study among subjects with stable CVD without known diabetes over a decade of follow-up are summarized as follows: Firstly, for FPG, IFG-ADA was associated with more than 60% higher risk of hard CVD events only in the age and sex adjusted model; however, NDM was associated with higher risk of recurrent CVD/CHD and their related hard outcomes, independent of traditional risk factors. Likewise, every 1.04 mmol/L increase in FPG was associated with 20, 27, 15 and 25% higher risk of CVD, hard CVD, CHD and hard CHD, respectively. Secondly, regarding 2 h-PCPG, subjects with NDM had 46% increased risk of CVD and CHD and 95% higher risk of hard CHD. Moreover, every 3.49 mmol/L increase in 2 h-PCPG was associated with 21% and 16% higher risk of CVD and CHD, respectively. Thirdly, FPG and 2 h-PCPG, whether as continuous or categorical variables were significant predictors of incident diabetes.

### Abnormal glucose tolerance and recurrent cardiovascular events

Impact of IFG, IGT and NDM on recurrent cardiovascular outcomes has been addressed in previous studies; however, two important issues should be noted: First, most of these studies were performed in the hospital setting among patients with a high baseline risk for recurrent events i.e., those with MI [11, 12, 29–33], or PCI/CABG [10]. Second, there were great differences between studies in terms of sample size, follow-up duration, approaching FPG and 2 h-PCPG as a continuous or categorical variable and heterogeneity in outcome definitions (CVD, MACE, all-cause or cardiovascular mortality). To our knowledge, the present study is the first population-based cohort with a long-term follow-up to investigate these associations in a heterogenic and relatively low risk population with stable CVD.

Findings of previous studies regarding the association of FPG with recurrent CVD were inconsistent. FPG as a continuous variable was not associated with recurrent CV outcomes [9, 10, 34] or had a borderline lower risk of recurrent CVD (0.85, 0.71–1.01, *p* = 0.06) [30, 35]; whereas each 1 mmol/L increase in FPG was associated with higher risk of MACE (28%) and cardiovascular mortality (51%) among post-MI patients in UK, hazards were not significant in the models including both FPG and 2 h-PCPG [36]. Our results showed that increasing level of FPG was significantly associated with higher risk of CVD/CHD; however, when both FPG and 2 h-PCPG were included in the model, no association was demonstrated.

With regards to the use of standard cut-offs among patients with prevalent CAD, IFG-WHO was not associated with MACE in the study by Tamita [HR 1.86 (0.86–3.87)] [12], any cardiac outcomes in the ARTEMIS study [9] and composite endpoints (including cardiovascular mortality, non-fatal MI, stroke, or hospitalization for heart failure) in the EUROASPIRE IV study [10]. Furthermore, in a population-based study among Japanese men, borderline hyperglycemia (FPG = 5.6–6.9 mmol/L) was not associated with recurrent CVD outcomes among those with prior CAD [37]. Similarly, prediabetes (using FPG and HbA1C criteria) was not associated with CV outcomes in Chinese patients [38, 39]. Besides, Lenzen et al. [40] revealed that abnormal glucose regulation (AGR) (IFG and IGT) was not an independent predictor for hard CV outcomes in the hospital-based setting while AGR in men with HF was associated with significant higher risk of recurrent CV outcomes [13]. The Otten et al. [41] study showed that IFG-ADA was associated with a hazard of 1.66 (1.05–2.61) for MACE. Based on our findings, among patients with stable CVD at an outpatient setting, IFG-ADA showed a signal for the association with hard CVD outcome, the value which did not reach to the significant level. Importantly, we have recently reported that the significant risk of IFG for CVD events in general population is attributable to those who converted from the IFG state to diabetes [42]; unfortunately, the current study did not have an adequate power to test this possibility in a cohort of subjects with previous CVD.

Focusing on 2 h-PCPG, some studies suggest that 2 h-PCPG is a better determinant to assess the prognosis of post ACS patients compared to FPG. Notably, some authors believe that adding 2 h-PCPG (but not FPG) to the Global Registry of Acute Coronary Events (GRACE) score (an established risk model for recurrent cardiac events), can improve its prediction power in post MI patients without known diabetes [30, 35]. Similarly, Chattopadhyay et al. showed that 2 h-PCPG is a better predictor of adverse post MI outcomes compared to FPG [36]. In this regard, our study showed a significant positive association between 2 h-PCPG and recurrent CVD/CHD outcomes. In some but not all studies, IGT was associated with worse post MI prognosis [10, 30, 34]. George et al. revealed that IGT is associated with higher risk of MACE but not hard CVD outcomes [31]. In line with studies among low risk population with prevalent CAD [9, 43], we also found no risk of IGT for recurrent CVD/CHD.

Regarding NDM, we found that NDM using FPG criteria was associated with CVD/CHD and their related hard outcomes. Among Chinese patients who underwent PCI, NDM (using FPG or HbA1C criteria) was an independent risk factor for MACE but not hard outcomes [38]. In the George et al. study, NDM (using FPG and/or 2 h-PCPG criteria) was associated with CVD and related hard outcomes [31]. However, in large studies conducted on European populations [10, 40], NDM was not associated with CVD outcomes. Using 2 h-PCPG criteria for definition of NDM, most studies showed that NDM [29, 34] or AGT (NDM plus IGT) are independently associated with higher risk of different CV outcomes [10–12, 32–34]; these findings are in agreement with ours indicating significant associations of NDM using 2 h-PCPG criteria with CVD, CHD and hard CHD.

### Incident diabetes outcome

History of CVD is known as a risk factor of incident diabetes among overweight and obese population [28]; however, it was not an independent risk factor for incident diabetes among Iranian population [44]. The present study showed that both FPG and 2 h-PCPG are strong independent predictors of incident diabetes. While in the EUROASPIRE IV study, 2 h-PCPG but not FPG, was a significant predictor [10], in the ARTEMIS study [9], both IGT and IFG groups had similarly higher risk for incident diabetes compared with the normoglycemia group.

## Strengths and limitations

Strengths of the current study are its prospective, longitudinal design with a long-term follow-up, reliable measurements of different covariates and careful adjustment for potential confounders. Moreover, our study included a heterogenous group of subjects with history of CAD in the stable phase of the disease and evaluated a wide range of outcomes including hard CVD/CHD events. The study limitations should also be considered: Firstly, we did not have data to calculate GRACE score including the ejection fraction of subjects; however, available variables of this score system such as heart rate were included in the multivariable model. Secondly, serum HbA1c levels were not available which may cause misclassification and underestimation of the risk associated with prediabetes; however, some well-known cohorts including Framingham Offspring Study have investigated associations of glycemic states with CV outcomes without HbA1c measurement [45]. Third, in our population based study, routine cardiac biomarkers were applied for diagnosis of MI (see definition of terms) in general hospitals. However, other promising investigational biomarkers such as high sensitivity c-Tn (hs-cTn), plasma asymmetric dimethylarginine (ADMA) and heart-type fatty acid binding protein (H-FABP) with a potential role in diagnosis of ACS [46, 47] or in the pathogenesis of restenosis were not assessed [48]. Fourth, data about diet were not available at the baseline recruitment of the study. Finally, this study was conducted on an Iranian urban population and the findings cannot be extrapolated to the rural areas.

## Conclusions

Among subjects with stable CVD, although increasing levels of FPG and 2 h-PCPG were associated with significant risk of recurrent CVD/CHD, only NDM but not prediabetes status was a significant risk factor for recurrent events. Moreover, FPG and 2 h-PCPG, as either continuous or categorical variables were significantly associated with incident diabetes.

## Supplementary Information

**Additional file 1: Table S1.** Risks of adverse cardiovascular outcomes based on different glucose intolerance categories. (complete case, n=301). ** Table S2. ** Adjusted HR (95% CI) for adverse cardiovascular outcomes per 1-SD increase of FPG and 2h-PCPG. (complete case, n=301). **Table S3.** Risks of adverse cardiovascular outcomes based on different glucose intolerance categories. (after excluding stroke from CVD definition, n=352).** Table S4.** Adjusted HRs (95% CI) for adverse cardiovascular events per 1-SD increase of FPG and 2 h-PCPG. (after excluding stroke from CVD definition, n=352). **Table S5.** Adjusted HR (95% CI) for adverse cardiovascular outcomes per 1-SD increase of FPG and 2h-PCPG among those with definite CAD. (n=164).

## Data Availability

All data and materials are available upon request.

## Abbreviations

CVD: Cardiovascular disease
CHD: Coronary heart disease
NFG: Normal fasting glucose
NGT: Normal glucose tolerance
IFG: Impaired fasting glucose
IGT: Impaired glucose tolerance
NDM: Newly diagnosed diabetes,
HR: Hazard ratio
CI: Confidence interval
SD: Standard deviation
MENA: Middle East and North Africa
TLGS: Tehran Lipid and Glucose Study
FPG: Fasting plasma glucose
2 h-PCPG: 2-H post challenge plasma glucose
BMI: Body mass index
WC: Waist circumference
SBP: Systolic blood pressure
DBP: Diastolic blood pressure,
TC: Total cholesterol,
HDL-C: High density lipoprotein-cholesterol
ECG: Electrocardiogram
ADA: American Diabetes Association
WHO: World Health Organization
MI: Myocardial infarction
