# Supplementary material for: Long term prognostic implication of newly detected abnormal glucose tolerance among patients with stable cardiovascular disease: a population-based cohort study
Source: J Transl Med. 2021 Jun 30;19:277. doi: 10.1186/s12967-021-02950-y (PMC8243871; doi:10.1186/s12967-021-02950-y)
Supplement: Supplementary file 1 — Additional file 1: Table S1. Risks of adverse cardiovascular outcomes based on different glucose intolerance categories. (complete case, n=301). Table S2. Adjusted HR (95% CI) for adverse cardiovascular outcomes per 1-SD increase of FPG and 2h-PCPG. (complete case, n=301). Table S3. Risks of adverse cardiovascular outcomes based on different glucose intolerance categories. (after excluding stroke from CVD definition, n=352). Table S4. Adjusted HRs (95% CI) for adverse cardiovascular events per 1-SD increase of FPG and 2 h-PCPG. (after excluding stroke from CVD definition, n=352). Table S5. Adjusted HR (95% CI) for adverse cardiovascular outcomes per 1-SD increase of FPG and 2h-PCPG among those with definite CAD. (n=164). [file 12967_2021_2950_MOESM1_ESM.docx]

| **Supplementary Table 1. Risks of adverse cardiovascular outcomes based on different glucose intolerance categories. (complete case, n=301)** | | | | | | | | | | | | |
| --- | --- | --- | --- | --- | --- | --- | --- | --- | --- | --- | --- | --- |
|  | | CVD  HR (95%CI) |  |  | Hard CVD  HR (95%CI) |  |  | CHD  HR (95%CI) |  |  | Hard CHD  HR (95%CI) |  |
|  | | Model 1 | Model 2 |  | Model 1 | Model 2 |  | Model 1 | Model 2 |  | Model 1 | Model 2 |
| **FPG-WHO (mmol/L)** | |  |  |  |  |  |  |  |  |  |  |  |
|  | <6.1 | 1.00 | 1.00 |  | 1.00 | 1.00 |  | 1.00 | 1.00 |  | 1.00 | 1.00 |
|  | 6.1-6.9 | 0.97(0.58-1.64) | 0.95(0.56-1.62) |  | 1.25(0.57-2.78) | 1.15(0.51-2.59) |  | 0.97(0.56-1.65) | 0.93(0.54-1.62) |  | 1.40(0.54-3.61) | 1.19(0.45-3.15) |
|  | ≥7 | 2.14(1.24-3.68) | 1.99(1.13-3.50) |  | 1.18(0.42-3.29) | 1.26(0.43-3.70) |  | 2.32(1.34-4.00) | 2.17(1.23-3.84) |  | 1.79(0.63-5.10) | 2.07(0.67-6.39) |
| **FPG-ADA (mmol/L)** | |  |  |  |  |  |  |  |  |  |  |  |
|  | <5.6 | 1.00 | 1.00 |  | 1.00 | 1.00 |  | 1.00 | 1.00 |  | 1.00 | 1.00 |
|  | 5.6-6.9 | 1.03(0.73-1.48) | 1.01(0.70-1.45) |  | 1.51(0.86-2.63) | 1.44(0.81-2.54) |  | 1.00(0.69-1.45) | 0.97(0.66-1.42) |  | 1.34(0.67-2.71) | 1.38(0.67-2.85) |
|  | ≥7 | 2.17(1.25-3.75) | 2.01(1.13-3.58) |  | 1.31(0.46-3.71) | 1.42(0.48-4.25) |  | 2.33(1.34-4.04) | 2.17(1.21-3.89) |  | 1.89(0.65-5.47) | 2.26(0.72-7.15) |
| **2h-PCPG (mmol/L)** | |  |  |  |  |  |  |  |  |  |  |  |
|  | <7.8 | 1.00 | 1.00 |  | 1.00 | 1.00 |  | 1.00 | 1.00 |  | 1.00 | 1.00 |
|  | 7.8-11.0 | 1.21(0.84-1.75) | 1.16(0.78-1.72) |  | 0.78(0.40-1.52) | 0.80(0.40-1.62) |  | 1.16(0.79-1.70) | 1.10(0.73-1.65) |  | 0.64(0.26-1.55) | 0.62(0.25-1.58) |
|  | ≥11 | 1.37(0.87-2.15) | 1.37(0.86-2.18) |  | 1.17(0.54-2.51) | 1.27(0.57-2.83) |  | 1.43(0.90-2.26) | 1.42(0.88-2.29) |  | 1.58(0.68-3.66) | 1.90(0.78-4.65) |
| CVD, cardiovascular disease; CHD, coronary heart disease; HR, hazard ratio; CI, confidence interval; FPG, fasting plasma glucose; 2h-PCPG, 2h post challenge plasma glucose; WHO, World Health Organization; ADA, American Diabetes Association.  Model 1: age and sex  Model 2: model 1+ body mass index, current smoking, family history of premature CVD, hypertension, hypercholesterolemia, low HDL-C, heart rate, use of aspirin, use of beta blockers and low physical activity. | | | | | | | | | | | | |

| **Supplementary Table 2. Adjusted HR (95% CI) for adverse cardiovascular outcomes per 1-SD increase of FPG and 2h-PCPG. (complete case, n=301)** | | | | | | | | |
| --- | --- | --- | --- | --- | --- | --- | --- | --- |
|  | CVD |  | Hard CVD |  | CHD |  | Hard CHD |  |
|  | HR (95%CI) | P-value | HR (95%CI) | P-value | HR (95%CI) | P-value | HR (95%CI) | P-value |
| **FPG** |  |  |  |  |  |  |  |  |
| Model 1 | 1.17(1.01-1.34) | 0.03 | 1.05(0.83-1.33) | 0.69 | 1.18(1.03-1.36) | 0.02 | 1.10(0.83-1.45) | 0.49 |
| Model 2 | 1.16(1.00-1.34) | 0.05 | 1.07(0.84-1.37) | 0.58 | 1.17(1.01-1.36) | 0.04 | 1.14(0.85-1.52) | 0.37 |
| **2h-PCPG** |  |  |  |  |  |  |  |  |
| Model 1 | 1.20(1.03-1.40) | 0.02 | 0.98(0.74-1.28) | 0.87 | 1.22(1.05-1.43) | 0.01 | 1.08(0.80-1.46) | 0.62 |
| Model 2 | 1.20(1.02-1.41) | 0.02 | 0.99(0.74-1.32) | 0.94 | 1.22(1.04-1.44) | 0.01 | 1.11(0.80-1.54) | 0.53 |
| CVD, cardiovascular disease; CHD, coronary heart disease; HR, hazard ratio; CI, confidence interval; FPG, fasting plasma glucose; 2h-PCPG, 2-h post challenge plasma glucose.  Model 1: age and sex  Model 2: model 1+ BMI, current smoking, family history of premature CVD, hypertension, hypercholesterolemia, low HDL-C, heart rate, use of aspirin, use of beta blockers and low physical activity. | | | | | | | | |

| **Supplementary Table 3. Risks of adverse cardiovascular outcomes based on different glucose intolerance categories. (after excluding stroke from CVD definition, n=352)** | | | | | | | | | | | | | |
| --- | --- | --- | --- | --- | --- | --- | --- | --- | --- | --- | --- | --- | --- |
|  | |  | CVD  HR (95%CI) | |  | Hard CVD  HR (95%CI) | |  | CHD  HR (95%CI) | |  | Hard CHD  HR (95%CI) | |
|  |  |  | Model 1 | Model 2 |  | Model 1 | Model 2 |  | Model1 | Model 2 |  | Model 1 | Model 2 |
| **FPG-WHO** **(mmol/L)** | | | |  |  |  |  |  |  |  |  |  |  |
|  | <6.1 |  | 1.00 | 1.00 |  | 1.00 | 1.00 |  | 1.00 | 1.00 |  | 1.00 | 1.00 |
|  | 6.1-6.9 |  | 0.99(0.62-1.58) | 0.93(0.57-1.49) |  | 1.30(0.64-2.64) | 1.12(0.54-2.33) |  | 0.97(0.60-1.56) | 0.91(0.56-1.48) |  | 1.37(0.61-3.09) | 1.19(0.52-2.72) |
|  | ≥7 |  | 2.07(1.35-3.18) | 2.08(1.32-3.27) |  | 1.57(0.74-3.31) | 1.80(0.81-3.97) |  | 1.84(1.18-2.89) | 1.87(1.17-3.00) |  | 1.54(0.65-3.66) | 1.85(0.73-4.69) |
| **FPG-ADA (mmol/L)** | | | |  |  |  |  |  |  |  |  |  |  |
|  | <5.6 |  | 1.00 | 1.00 |  | 1.00 | 1.00 |  | 1.00 | 1.00 |  | 1.00 | 1.00 |
|  | 5.6-6.9 |  | 1.12(0.81-1.55) | 1.08(0.77-1.51) |  | 1.89(1.13-3.14) | 1.76(1.03-2.99) |  | 1.12(0.80-1.56) | 1.09(0.77-1.53) |  | 1.55(0.85-2.84) | 1.51 (0.81-2.83) |
|  | ≥7 |  | 2.15(1.38-3.34) | 2.16(1.35-3.45) |  | 1.92(0.89-4.17) | 2.27(0.99-5.20) |  | 1.92(1.22-3.04) | 1.96(1.20-3.18) |  | 1.73(0.71-4.21) | 2.16(0.83-5.65) |
| **2h-PCPG** **(mmol/L)** | | | |  |  |  |  |  |  |  |  |  |  |
|  | <7.8 |  | 1.00 | 1.00 |  | 1.00 | 1.00 |  | 1.00 | 1.00 |  | 1.00 | 1.00 |
|  | 7.8-11.0 |  | 1.01(0.72-1.43) | 0.95(0.67-1.36) |  | 0.88(0.48-1.61) | 0.81(0.43-1.52) |  | 1.05(0.74-1.49) | 1.01(0.70-1.45) |  | 1.02(0.52-2.02) | 1.00(0.49-2.04) |
|  | ≥11 |  | 1.52(1.05-2.22) | 1.50(1.02-2.21) |  | 1.58(0.87-2.89) | 1.62(0.85-3.08) |  | 1.44(0.97-2.12) | 1.44(0.96-2.16) |  | 1.64(0.81-3.34) | 1.84(0.86-3.96) |
| CVD, cardiovascular disease; CHD, coronary heart disease; HR, hazard ratio; CI, confidence interval; FPG, fasting plasma glucose; 2h-PCPG, 2h post challenge plasma glucose; WHO, World Health Organization; ADA, American Diabetes Association.  Model 1: age and sex  Model 2: model 1+ body mass index, current smoking, family history of premature CVD, hypertension, hypercholesterolemia, low HDL-C, heart rate, use of aspirin, use of beta blockers and low physical activity. | | | | | | | | | | | | | |

| **Supplementary Table 4. Adjusted HRs (95% CI) for adverse cardiovascular events per 1-SD increase of FPG and 2 h-PCPG. (after excluding stroke from CVD definition, n=352)** | | | | | | | | |
| --- | --- | --- | --- | --- | --- | --- | --- | --- |
|  | CVD |  | Hard CVD |  | CHD |  | Hard CHD |  |
|  | HR (95%CI) | P-value | HR (95%CI) | P-value | HR (95%CI) | P-value | HR (95%CI) | P-value |
| **FPG** |  |  |  |  |  |  |  |  |
| Model 1 | 1.20(1.07-1.34) | 0.001 | 1.22 (1.02-1.47) | 0.03 | 1.14(1.02-1.28) | 0.02 | 1.13(0.88-1.44) | 0.34 |
| Model 2 | 1.19(1.06-1.33) | 0.004 | 1.24(1.02-1.52) | 0.03 | 1.13(1.00-1.27) | 0.05 | 1.16(0.89-1.50) | 0.27 |
| **2h-PCPG** |  |  |  |  |  |  |  |  |
| Model 1 | 1.21(1.07-1.37) | 0.002 | 1.17(0.94-1.45) | 0.15 | 1.16(1.02-1.32) | 0.02 | 1.12(0.86-1.45) | 0.39 |
| Model 2 | 1.20(1.06-1.36) | 0.005 | 1.19(0.94-1.50) | 0.15 | 1.15(1.01-1.31) | 0.03 | 1.16(0.88-1.54) | 0.30 |
| CVD, cardiovascular disease; CHD, coronary heart disease; HR, hazard ratio; CI, confidence interval; FPG, fasting plasma glucose; 2h-PCPG, 2-h post challenge plasma glucose.  Model 1: age and sex  Model 2: model 1+ body mass index, current smoking, family history of premature CVD, hypertension, hypercholesterolemia, low HDL-C, heart rate, use of aspirin, use of beta blockers and low physical activity. | | | | | | | | |

| **Supplementary Table 5. Adjusted HR (95% CI) for adverse cardiovascular outcomes per 1-SD increase of FPG and 2h-PCPG among those with definite CAD. (n=164)** | | | | | | | | |
| --- | --- | --- | --- | --- | --- | --- | --- | --- |
|  | CVD |  | Hard CVD |  | CHD |  | Hard CHD |  |
|  | HR (95%CI) | P-value | HR (95%CI) | P-value | HR (95%CI) | P-value | HR (95%CI) | P-value |
| **FPG** |  |  |  |  |  |  |  |  |
| Model 1 | 1.30(1.14-1.49) | <0.001 | 1.40(1.16-1.70) | 0.001 | 1.19(1.03-1.37) | 0.02 | 1.30(1.01-1.68) | 0.04 |
| Model 2 | 1.32(1.13-1.53) | <0.001 | 1.66(1.31-2.01) | <0.001 | 1.18(1.00-1.40) | 0.05 | 1.50(1.11-2.03) | 0.009 |
| **2h-PCPG** |  |  |  |  |  |  |  |  |
| Model 1 | 1.33(1.14-1.56) | <0.001 | 1.36(1.06-1.76) | 0.02 | 1.22(1.03-1.44) | 0.02 | 1.30(0.95-1.78) | 0.10 |
| Model 2 | 1.32(1.10-1.57) | 0.002 | 1.59(1.19-2.11) | 0.001 | 1.19(0.99-1.44) | 0.07 | 1.49(1.04-2.13) | 0.02 |
| CVD, cardiovascular disease; CHD, coronary heart disease; HR, hazard ratio; CI, confidence interval; FPG, fasting plasma glucose; 2h-PCPG, 2h post challenge plasma glucose.  Model 1: age and sex  Model 2: model 1+ body mass index, current smoking, family history of premature CVD, hypertension, hypercholesterolemia, low HDL-C, heart rate, use of aspirin, use of beta blockers and low physical activity. | | | | | | | | |
